# Congenital Alveolar Fissure, with Its Accompanying Dental Condition

**Published:** 1885-04

**Authors:** Oakley Coles


ARTICLE III.

### CONGENITAL ALVEOLAR FISSURE, WITH ITS ACCOMPANYING DENTAL CONDITION.

BY OAKLEY COLES, L. D. S. ENG.

It is proposed in the present paper to deal with the consideration of 31 cases of congenital fissure of the alveoli and hard palate, occuring in my own practice.

The numbers given will correspond with numbers attached to the models.

Following the example of Dr. Albrecht and Professor Turner, I shall speak of the tooth placed anteriorly to the true canine on the side where the fissure occurs as the pre-canine tooth, though I do not by this wish to admit the acceptance of the theory that man has ever had, normally, six true incisor teeth. For simplification of comparison I shall follow the order taken in the valuable paper by Professor Turner, and describe first, left alveolar fissure; secondly, right alveolar fissure; and thirdly, double alveolar fissure. I shall endeavor, as far as possible, to avoid coming to any definite conclusions. The views put forward are so interesting and yet so greatly at variance with much that has hitherto been held as a satisfactory explanation of the origin of fissure in the alveolar region, that it seems to me desirable we should for the present content ourselves with the accumulation of facts, rather than attempt to settle the question at issue by any premature generalization.

In speaking of right and left I shall always refer to that of the patient and not of the onlooker.

### FIRST SERIES—LEFT-SIDED FISSURED ALVEOLI.

Model 11. Case 1.—Left-sided alveolar fissure, permanent central incisors, and first permanent molars erupted. Four temporary pre-molars, and two temporary canines in normal position, no evidence of temporary or permanent lateral on right side, small pre-canine in process of eruption in the left maxillary bone.

Model 22. Case 2.—First permanent molars, right permanent central and lateral incisors erupted, four temporary molars and two canines persistent, pre-canine partially erupted on left side.

Model 16. Case 3.—Dentition transitional, well erupted pre-canine on left side.

Model 21. Case 4.—Permanent molars and bicuspids erupted, right central and lateral incisor and canine in position, left apparently temporary, canine persistent, well developed pre-canine, no evidence of left central incisor.

Model 27. Case 5.—Dentition transitional, first right and left bicuspids in process of eruption, right and left canine in process of eruption, right and left central well developed, right lateral rudimentary in form, no evidence of pre-canine on left side.

Model 18. Case 6.—Adult dentition, two bicuspids and canine on left side normal in form and position, well marked pre-canine on left side. The dentition on the right side apparently irregular in character.

Model 26. Case 7.—Adult normal dentition, well marked pre-canine on left side.

Model 19. Case 8.—Adult normal dentition, crown of pre-canine on left side apparently excised.

Model 13. Case 9.—Adult dentition, no pre-canine on left side, and no left central incisor.

Model 23. Case 10.—Adult dentition, no pre-canine on left side and no left central incisor.

Model 17. Case 11.—Adult dentition, no canine or pre-canine on left side, and no left central incisor.

Model 14. Case 12.—Immature dentition, no right canine, indications of left canine and pre-canine, in process of eruption.

Model 28. Case 13—Adult dentition, no pre-canine on left side, and no left central incisor.

Model 29. Case 14.—Adult dentition, imperfectly formed teeth, no pre-canine on left side, and no left central incisor.

Model 15. Case 15.—Immature dentition, no pre-canine, permanent canine apparently in process of eruption.

Model 24. Case 16.—Adult dentition, indications of two teeth on the left side in the canine region.

Model 20. Case 17.—Adult dentition, canine on left side, no indication of pre-canine.

### SECOND SERIES—RIGHT-SIDED FISSURED ALVEOLI.

Model 1. Case 18.—Adult dentition, canine on right side, pre-canine erupted in the palate, but at the margin of the cleft no right central incisor, and one bicuspid missing on each side.

Model 8. Case 19.—Adult dentition, canine on right side, pre-canine erupted in the palate, but at the margin of the cleft, right central incisor in normal position.

Model 9. Case 20.—Adult dentition, canine on right side, pre-canine erupted in the palate at the margin of the cleft, right central present but semi-rotated.

Model 6. Case 21.—Adult dentition, canine on right side, no pre-canine, no right central incisor, left central incisor rudimentary in character.

Model 10. Case 22.—Adult dentition, canine on right side, pre-canine uncertain, right central present.

Model 2. Case 23.—Adult dentition, canine on right side, no pre-canine, no right central.

Model 3. Case 24.—Adult dentition, canine on right side, pre-canine excised, erupted in the palate at the margin of the cleft, no right central incisor, two bicuspids missing.

Model 7. Case 25.—Adult dentition, canine on right side, pre-canine possibly in the gum, right central incisor present.

Model 4. Case 26.—Adult dentition, neither canine nor pre-canine erupted, no right central, left central excised.

Model 5. Case 27.—Adult dentition, canine on right side, no pre-canine, no right central, left lateral apparently extracted from outside of dental arch.

THIRD SERIES.—DOUBLE ALVEOLAR FISSURE.

Model 30. Case 28.—Adult dentition, canine on each side in normal position, pre-canine on right side normally placed in dental arch, pre-canine on left side erupting outside of dental arch.

Model 23. Case 29.—Adult dentition, canine on each side, pre-canine on left side in dental arch but not fully erupted, pre canine on right side imperfectly erupted outside dental arch.

Model 32. Case 30.—Adult dentition, canine on each side, pre-canine on left side fully erupted in dental arch, condition of right side uncertain.

Model 31. Case 31.—Adult dentition, canine on each side, no pre-canine.

### OBSERVATIONS.

1. It will be observed that there is not a single instance of fissure of the alveoli occuring between a true lateral incisor and canine on either side of the mouth.

2. That in five out of ten cases of right-sided fissure the right central incisor is missing.

3. That in eight out of seventeen cases of left-sided fissure the left central incisor is missing.

4. That in five out of seventeen cases of left-sided fissure a pre-canine tooth is clearly present.

5. That in four out of ten cases of right-sided fissure the pre-canine is clearly present.

6. That in two out of four cases of double alveolar fissure a pre-canine is present on each side, and in a third case, on the left side.

7. That there is not any sufficient evidence in any case of increase in the number of teeth in the pre-canine region, whilst there is distinct evidence in some cases of the reverse condition, and also of imperfect development.

8. The evidence at present before us is quite insufficient for the purpose of arriving at any conclusion, whilst the seven models without any numbers attached will indicate the singular liabillity of the pre-canine region of the upper jaw to irregularity, of a purely dental origin.

9. The chief aims of observers for some time to come must be the examination of fissured alveoli very early in life, the careful dissection of the premaxillary bone in cases where it has been excised for the better treatment of hare-lip during infancy and the collection of very accurate and carefully preserved models for the purpose of recording the exact condition of the teeth at various ages.—*London Dental Record.*

